# Using Jupyter Notebooks for re-training machine learning models

**DOI:** 10.1186/s13321-022-00635-2

**Published:** 2022-08-13

**Authors:** Aljoša Smajić, Melanie Grandits, Gerhard F. Ecker

**Affiliations:** Department of Pharmaceutical Sciences, University of Vienna, Vienna, Austria

**Keywords:** Classification models, Transporter proteins, Decentralization, Re-training, Jupyter Notebook

## Abstract

Machine learning (ML) models require an extensive, user-driven selection of molecular descriptors in order to learn from chemical structures to predict actives and inactives with a high reliability. In addition, privacy concerns often restrict the access to sufficient data, leading to models with a narrow chemical space. Therefore, we propose a framework of re-trainable models that can be transferred from one local instance to another, and further allow a less extensive descriptor selection. The models are shared via a Jupyter Notebook, allowing the evaluation and implementation of a broader chemical space by keeping most of the tunable parameters pre-defined. This enables the models to be updated in a decentralized, facile, and fast manner. Herein, the method was evaluated with six transporter datasets (BCRP, BSEP, OATP1B1, OATP1B3, MRP3, P-gp), which revealed the general applicability of this approach.

## Introduction

The importance of machine learning (ML) approaches in drug discovery and in silico toxicity prediction has shown a significant increase in recent years. As available toxicity data has significantly increased [1–3], ML approaches became an essential part of the drug discovery pipeline. Public–private partnerships such as eTOX [4] and eTRANSAFE [5], as well as public databases (ChEMBL [6], PubChem [7]) enable trustful data supply for the establishment of predictive ML models. For training and improving the performances of ML models, a large amount of data is crucial [8]. However, when seeking to pool data from multiple sources, multiple restrictions occur. Companies quite often restrict access to in house data due to their business value. In addition, collecting, curating, and preserving data requires a lot of effort and time.

Furthermore, once a sufficient amount of qualitative data is established, additional challenges can occur on the path towards the creation of efficient ML models. The selection of chemical descriptors best suited to derive models of sufficient quality is one of them. The selection of a proper set of descriptors is an extensive, time-intensive, and still mostly manual process, especially when trying to understand relationships between chemical properties and their effect on biological targets [9]. Depending on the biological target, the descriptors best suited can considerably vary. Combined with the fact that additional hyper-parameters have to be tuned for each model, the creation of high accuracy ML models becomes an exhaustive process.

To overcome these issues and allow the user to establish predictive models in an easy and fast way, we created a framework that can be used in a semi-automated fashion for the creation and/or re-training of ML models for predicting inhibitory activity towards ABC and SLC transporters. Furthermore, in comparison to previous methods our approach does not require descriptor selection and hyperparameter search which enables fast and efficient model building.

A set of transporters, mainly used in this study, has caught the attention of regulatory agencies such as FDA, EMA, and the Japanese regulatory agency, as the inhibition of these proteins may play a role in drug-drug interactions and/or drug-induced liver injury. Therefore, the prediction of inhibitory profiles of small molecules towards these set of transporters can help to guide safety assessments of new drugs as often requested or recommended by regulatory agencies. Additionally, the knowledge can further help in terms of prioritization of compounds at the early Drug Discovery stage by medicinal chemists [10–17].

Combining Jupyter Notebooks (JNs) [18] as a framework for creating ML models and high-quality data regarding transport membrane proteins to train these models, shareable models can be built for the assessment of compounds for their interaction profile. In general, JN is a web-based interactive computing platform that enables the combination of computer code (e.g. python) and rich text elements (e.g. figures). A web browser is used to navigate in the JN app, and the established graphical user interface allows a better representation of files and so-called notebook documents. These notebook documents can be executed as well as read by users, as they contain code, rich text, images, plots, interactive figures and widgets. These notebooks can be easily shared since they are saved as structured text files (JSON format) and enable the transfer of the code of the model from one instance to another for re-training the model [19]. This allows the enrichment of the chemical space of the model. The notebook further provides a generalizable set of molecular descriptors for the ABC and SLC transporter families that has been shown to be applicable at least for the transporter proteins BCRP, BSEP, OATP1B1, OATP1B3, MRP3, and P-gp. The procedure was selected as it comprises the possibility of sharing the notebook in a facile manner and the creation of workflows for non-experienced users. By uploading data to the JN, the code can be executed which will allow the creation of models and the verification of the models within the JN. In addition, due to the ease of the integration of RDKit, JNs comprise a versatile tool for cheminformatics tasks.

Subsequently, JNs are great tools for educational purposes. The TeachOpenCADD platform by AG Volkamer has demonstrated this by creating JNs with step-by-step tutorials that can be used as a teaching platform for classroom lessons and self-studying. Open-source data and Python packages are used as tools for establishing both ligand- and structure-based approaches. The usage of these JNs provides knowledge in the field of cheminformatics and structural bioinformatics for students and users interested in these topics [20]. Therefore, our JN not only offers the possibility of improving the ML models, model building and predictions for the six endpoints but also offers students, universities and interested users to learn more about model building, data handling, datasets, standardization procedure, descriptor calculation and model evaluation in cheminformatics.

## Methods

### Dataset preparation

In this study, datasets of six different transmembrane transport proteins (BCRP, BSEP, OATP1B1, OATP1B3, MRP3, P-gp) were used as a case study [21–46]. Firstly, datasets from the Vienna LiverTox Workspace (LiverTox) [47] were chosen, as these datasets were already published and used for the development of predictive models. The corresponding web service allows the prediction of substrates and inhibitors for a set of ABC and SLC transporters.

Secondly, an in-house KNIME workflow was used for the retrieval of additional new data from public platforms such as ChEMBL and PubChem (ChEMBL26 [48], CheEMBL27 [49], ChEMBL28 [50], PubChem [7]). The data from ChEMBL 26 and 27 were used as additional training sets (see below), while data from ChEMBL 28 and additional data from Pubchem served as test sets. Activity values were taken from the original publication and class labeling for binary classification was applied based on a threshold of an IC50 value of 10 µM. All data sets were provided in sdf-format together with a binary classification (0/inactive or 1/active) for each of the six endpoints. For each compound the InChIs (IUPAC International Chemical Identifiers), InChI Keys and SMILES (Simplified Molecular Input Entry Specification) were calculated. All datasets are available on GitHub at https://github.com/PharminfoVienna/Retraining_Notebook/tree/main/data.

Before following the standardization protocol, stereochemistry information was removed from the InChIs and duplicated InChIs were identified. In case duplicates show the same class label, one of the compounds was kept. Otherwise, both compounds were removed. Data cleaning and standardization was performed using a modified version of the Standardizer provided by Atkinson (available at https://github.com/flatkinson/standardiser). This tool was applied to remove salts, neutralize, and discard non-organic compounds. Tables 1 and 2 show the number of data points available per transporter for LiverTox and for the newly collected datasets. The datasets were further used to generate classification models which allow the prediction of inhibitors for a number of liver transporters involved in severe side effects.

**Table 1:** Overview of the six transporter datasets which are provided on the LiverTox workspace

Endpoint	LiverTox training		LiverTox test	
	Actives	Inactives	Actives	Inactives
Breast cancer resistance protein (BCRP)	432	542	109	86
Bile salt export pump (BSEP)	114	410	43	116
Organic anion transporting polypeptide 1B1 (OATP1B1)	178	1472	64	137
Organic anion transporting polypeptide 1B3 (OATP1B3)	116	1547	40	169
Multidrug resistance associated protein (MRP3)	32	52	–	–
P-glycoprotein (Pgp)	612	549	86	48

**Table 2:** Overview of the six transporter datasets that were used for the training of the models

Endpoint	Training		Test	
	Actives	Inactives	Actives	Inactives
Breast cancer resistance protein (BCRP)	904	786	149	38
Bile salt export pump (BSEP)	221	1100	3	7
Organic anion transporting polypeptide 1B1 (OATP1B1)	292	1675	18	3
Organic anion transporting polypeptide 1B3 (OATP1B3)	168	1818	13	4
Multidrug resistance associated protein (MRP3)	74	569	0	3
P-glycoprotein (Pgp)	1281	953	136	236

The training set comprises data from LiverTox plus those extracted from ChEMBL 26 and 27, the test set contains data extracted from ChEMBL 28 and PubChem

### Descriptor selection

**Fig. 1:**
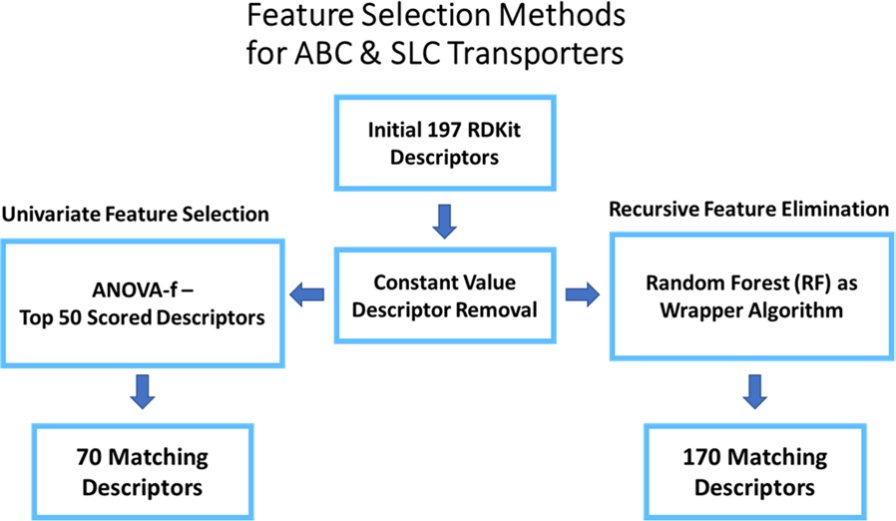
Schematic overview of the descriptor analysis carried out for both ABC and SLC transporters

**Fig. 2:**
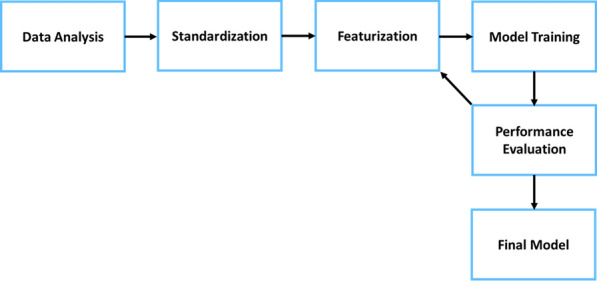
Graphical Illustration of the workflow for model generation

For the characterization of the chemical space related to ABC and SLC transporter inhibition, a variety of molecular descriptors from the RDKit library (version 2020.09.1) were used [51]. These molecular descriptors enable the translation of chemical structures into numerical representations of atomic or molecular properties of compounds. In total, 197 two-dimensional (2D) descriptors were chosen as a starting point for the selection of features applicable for ABC and SLC transporters. Herein, three different feature selection methods from the scikit-learn Python library (version 0.24.2) were applied: VarianceThreshold, Univariate feature selection, and Recursive feature elimination. By applying VarianceThreshold all calculated molecular descriptors with zero variance were removed. As a next step, best descriptors were selected based on a univariate statistical approach. ANOVA-f was chosen over mutual information due to the nature of the six transporter datasets. This method estimates the degree of linear dependency by using the F-test approach. In parallel, recursive feature elimination (RFE) was performed to select features by recursively considering subsets of molecular descriptors. A random forest wrapper was used for assigning the weights. As a last step, the results of both univariate feature selection and recursive feature elimination methods were compared from each dataset. Molecular descriptor results were then manually aligned with each other. 70 features were found to match within all transporters using the top 50 scored ANOVA-f method and 170 using the RFE method. The resulting 70 descriptors were used for the creation of the final models (see Fig. 1). A graphical representation of the workflow can be seen in Fig. 2.

### Model generation

Four different classifiers, namely logistic regression, support vector machine, random forest, and k-nearest neighbor were used for model generation. The scikit-learn Python library (version 0.24.2) implementations were used to train binary classification models for the six above mentioned datasets.

### Hyperparameter grid search

To find the optimal parameters for each classifier, a grid search of the hyperparameters was performed. The following parameters were used:

*Logistic regression:*

C: 0.01, 0.1, 0.2, 0.3, 0.4, 0.5, 1, 10, 50, 100, 1000

max_iter 1,10,100,100,1000,10000.

*Support vector machine:*

C: 0.01, 0.1, 1.0, kernel: linear.

C: 0.01, 0.1, 0.5, 1.0, 10.0, 50, 100, 1000, kernel: rbf,

C: 0.01, 0.1, 0.5, 1.0, 10, 50, 100, 1000, gamma: 0.0001, 0.001, 0.01, 0.1, 0.5, 1.0, 10.0, 50.0, 100.0, C: 0.0001, 0.001, 0.01, 0.1, 0.5, 1, 10, 50, 100, kernel: rbf

*Random forest:*

n_estimators: 10, 25, 50, 75, 100, 250, 500.

max_depth: 2, 3, 4, 6, 10, 15, 20.

*k-Nearest neighbor:*

n_neighbors: 3, 5, 9, 11, 13, 17, 19,

weights: uniform.

distance metric: Euclidean.

### Training procedure, cross-validation, and evaluation

In a first step, prediction models were generated simply based on the LiverTox dataset and the settings mentioned above. The performance of these models was compared with the ones obtained from the LiverTox models [47] to validate our approach.

In a next step, the newly collected datasets of the six transporters were used for the training of the actual models. The performance of the models was evaluated using a tenfold cross-validation, and the statistical metrics, such as accuracy, sensitivity, specificity, and balanced accuracy were calculated (see Table 3). For that, the scikit-learn Python library was used. Additionally, an external test set was used to test the new generated models. This test set was collected from ChEMBL28 and PubChem and only data which was novel to the training set was kept.

**Table 3:** Statistical metrics for all four models of each dataset

Models	LR		SVM		RF		k-NN	
	Train	Test	Train	Test	Train	Test	Train	Test
*BCRP*								
Accuracy	0.73	0.74	0.76	0.67	0.80	0.70	0.76	0.70
Sensitivity	0.75	0.81	0.75	0.69	0.79	0.71	0.79	0.74
Specificity	0.69	0.50	0.77	0.61	0.83	0.63	0.73	0.53
Balanced accuracy	0.72	0.65	0.76	0.65	0.80	0.67	0.76	0.63
F1-score	0.74	0.83	0.77	0.77	0.80	0.79	0.78	0.79
AUC	0.80	0.65	0.83	0.65	0.88	0.67	0.80	0.63
Precision	0.74	0.86	0.79	0.87	0.85	0.88	0.77	0.86
MCC	0.46	0.28	0.53	0.25	0.61	0.28	0.52	0.23
*BSEP*								
Accuracy	0.84		0.72	–	0.78	–	0.83	-
Sensitivity	0.22	–	0.84	–	0.79	–	0.52	-
Specificity	0.96	–	0.69	–	0.77	–	0.89	-
Balanced accuracy	0.59	–	0.77	–	0.77	–	0.71	-
F1-score	0.30	–	0.54	–	0.57	–	0.53	-
AUC	0.73	–	0.85	–	0.87	–	0.79	-
Precision	0.59	–	0.42	–	0.50	–	0.60	-
MCC	0.28	–	0.44	–	0.49	–	0.45	-
*OATP1B1*								
Accuracy	0.86	0.38	0.80	0.76	0.85	0.71	0.87	0.71
Sensitivity	0.20	0.33	0.74	0.83	0.63	0.72	0.35	0.67
Specificity	0.97	0.67	0.81	0.33	0.89	0.67	0.96	1
Balanced accuracy	0.59	0.50	0.77	0.58	0.74	0.69	0.65	0.83
F1-score	0.27	0.48	0.52	0.86	0.55	0.81	0.43	0.80
AUC	0.77	0.50	0.83	0.58	0.84	0.69	0.81	0.83
Precision	0.47	0.86	0.40	0.88	0.49	0.93	0.58	1
MCC	0.24	-	0.44	0.15	0.47	0.29	0.34	0.47
*OATP1B3*								
Accuracy	0.91	0.35	0.84	0.71	0.86	0.59	0.92	0.65
Sensitivity	0.14	0.23	0.81	0.77	0.77	0.69	0.36	0.62
Specificity	0.98	0.75	0.84	0.50	0.87	0.25	0.97	0.75
Balanced accuracy	0.56	0.49	0.83	0.64	0.82	0.47	0.67	0.68
F1-score	0.20	0.35	0.46	0.80	0.48	0.72	0.41	0.73
AUC	0.79	0.49	0.88	0.64	0.89	0.47	0.80	0.68
Precision	0.45	0.75	0.32	0.83	0.35	0.75	0.50	0.89
MCC	0.20	− 0.02	0.44	0.25	0.46	− 0.05	0.38	0.31
*MRP3*								
Accuracy	0.88	–	0.60	–	0.59	–	0.78	–
Sensitivity	0	–	0.77	–	0.68	–	0.20	–
Specificity	0.99	–	0.58	–	0.59	–	0.86	–
Balanced accuracy	0.5	–	0.67	–	0.62	–	0.53	–
F1-score	0	–	0.43	–	0.37	–	0.21	–
AUC	0.44	–	0.67	–	0.63	–	0.57	–
Precision	0.1	–	0.35	–	0.32	–	0.34	–
MCC	0	–	0.30	–	0.20	–	0.12	–
*P-gp*								
Accuracy	0.74	0.65	0.72	0.68	0.76	0.68	0.71	0.64
Sensitivity	0.81	0.92	0.72	0.81	0.81	0.92	0.76	0.88
Specificity	0.64	0.28	0.71	0.50	0.70	0.35	0.64	0.32
Balanced accuracy	0.73	0.60	0.71	0.65	0.76	0.64	0.70	0.60
F1-score	0.78	0.75	0.73	0.74	0.79	0.77	0.75	0.74
AUC	0.80	0.60	0.77	0.65	0.80	0.64	0.76	0.60
Precision	0.77	0.64	0.78	0.69	0.80	0.66	0.75	0.64
MCC	0.46	0.27	0.44	0.33	0.53	0.34	0.43	0.25

Test: External Dataset

*Train: tenfold cross-validation

## Applicability domain

Local outlier factor (LOF) as described by Breunig and coworkers was used for the calculation of the applicability domain [52] and as implemented in the scikit-learn Python library (version 0.24.2). In this approach the local densities of the nearest neighbors of a compound are compared to its local densities, and a factor from 0 to 1 is assigned. In brief, if the local density is greater or equal to its surrounding, a compound is considered inside the domain, otherwise it is considered outside the domain.

The following parameters were used:

- 5 nearest neighbors
- novelty = True
- Contamination = 0.1
- Euclidean metric
- Minmax scaled descriptors
- First two principal components were chosen as input

## Results

### Descriptor analysis

**Fig. 3:**
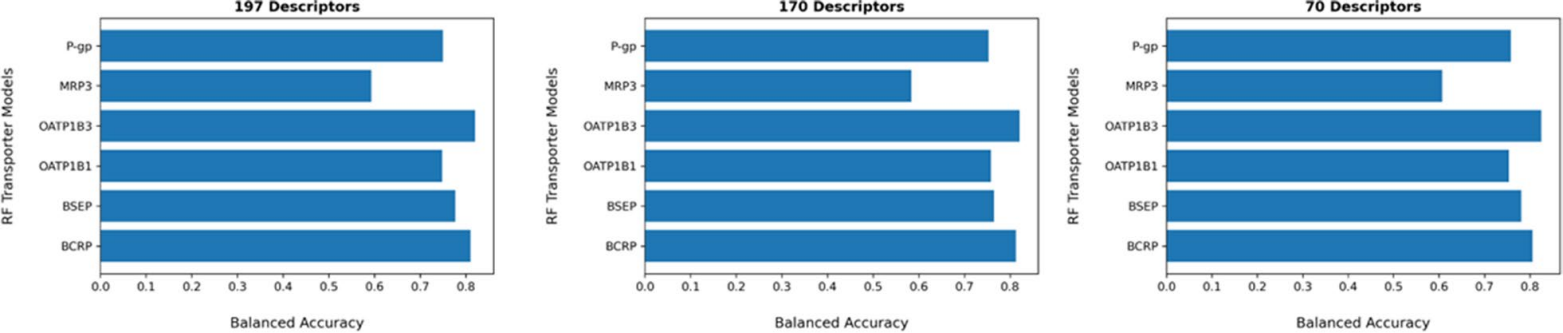
Comparison of the performances (balanced accuracy) of random forest models using 197, 170 and 70 descriptors

Three different feature selection methods were applied. Variance threshold setting to zero, univariate feature selection using ANOVA-f, and RFE with a random forest wrapper (default settings) were used for the retrieval of the most relevant molecular descriptors from the RDKit module for each dataset. However, once molecular descriptors with constant values were removed, the ANOVA-f and RFE method were applied. For the RFE method, different sets of descriptors were obtained for each transporter. The obtained descriptors were then aligned with each other for the identification of the most frequent descriptors occurring in each dataset. However, 170 descriptors were obtained, which is still considered as a high number considering the basic principle of parsimony in QSAR. Therefore, we conducted in parallel the ANOVA-f approach. Instead of using all scored molecular descriptors, we decided to use only the best 50 scored molecular descriptors for the alignment procedure. As our idea was to keep the number of descriptors as low as possible, we set the threshold to 50 for the alignment, as the performance of the models decreased in individual cases when a lower number was applied. The alignment of each set of resulted descriptors from the six transporter proteins was then conducted. This resulted in a final set of 70 descriptors. The impact of 197, 170 and 70 descriptors on all four models were then examined by calculating the balanced accuracy for each dataset. Interestingly, we obtained similar results using only 70 descriptors from the ANOVA-f approach (see Fig. 3) compared to 197 and 170 descriptors obtained from the RFE method. Therefore, we decided to implement the resulted 70 molecular descriptors retrieved from the ANOVA-f approach in the Jupyter Notebook.

### Performance of the ML models

For the development of predictive models that can be shared in an easy manner and used for all six transporter datasets, four distinct modeling strategies were applied. Logistic regression-, support vector machine-, random forest- and k-nearest neighbor classifiers were used to train models with the datasets from LiverTox. This concept was used to validate our approach to use it for the actual model generation. The comparison of the performance indicated similar results as shown in the documentation of LiverTox. The support vector and random forest models performed overall better. For the improvement of the models, new datasets for all six transporter datasets were collected from ChEMBL and PubChem. Further, newly published data from Chembl28 and PubChem were used as external datasets, whereas the previous versions were implemented for the training of the four modeling approaches. Again, the three feature selection methods and a hyperparameter search were conducted for the optimal number of descriptors and parameters. Finally, we obtained for each transporter two models, support vector and random forest, with a very similar balanced accuracy ranging from 0.67 till 0.83 for SVM and 0.62 till 0.82 for RF via tenfold cross validation within the various transporters. Overall, we observed that training the models with a subset of all descriptors, addition of new chemical space and the application of the grid search can improve the model performance as compared to the LiverTox models, especially considering the balanced accuracy.

## Discussion

Fast and facile model generation for binary classification tasks that are applicable for more than one transporter and additionally allow a retraining of the model is of great interest. Current ML model approaches that were developed are often based on one protein when trying to predict substances as transporter inhibitors or non-inhibitors [32, 53–55]. This makes it harder to generalize models when trying to predict substances for a group of transporters. As the selection of appropriate molecular descriptors can vary from one protein to another, this becomes a quite challenging step. Therefore, we established a Jupyter Notebook that allows the user to generate classification models for six transporter proteins (BCRP, BSEP, OATP1B1, OATP1B3, MRP3, P-gp) without intensive descriptor analysis and hyperparameter search. Moreover, these models can be shared between two instances for additional training. Our analysis indicated that 70 molecular descriptors from the RDKit module can be used for the creation of well-performing predictive models when random forest and support vector classifiers are used. The comparison of the feature selection methods implemented in the scikit-learn Python library showed to be useful for the reduction of descriptors by maintaining a good performance for most of the transporter models and establishing a general set of 70 descriptors for all six transporter proteins. However, in the case of MRP3 an overall low performance was obtained due to a low amount of available data points. The data gathering step revealed that only two transporter proteins, namely BCRP and P-gp, covered a well-balanced number of actives and inactives. This can be visualized when comparing the resulted precision with the remaining datasets that are unbalanced. Both, P-gp and BCRP models, predict correctly 76 to 80% of the cases when a random forest classifier was chosen. Interestingly, for all except MRP3, both good sensitivity and specificity values were retrieved, although the other transporters possess an unbalanced dataset. Only for OATP1B1 a sensitivity lower than 70% was obtained. Best performance was retrieved using the BCRP dataset with a balanced accuracy of 80%, precision of 85%, and sensitivity and specificity values from 79 to 83%. This can be explained by the high number of well-curated data points and the balanced number of actives and inactives in the dataset. Nevertheless, these models can be used for re-training and therefore the performance can increase once more data is available. For each transporter protein a tenfold cross-validation was performed and an external dataset was used for a thorough evaluation, after the final model was trained. A reasonable amount of test compounds was collected for BCRP and P-gp transporters. In the case of OATP1B1 and OATP1B3 more than 17 compounds were retrieved, and less than 10 compounds were obtained for BSEP and MRP3. Therefore, an external validation was meaningful when BCRP and P-gp test sets were evaluated. In both cases, the balanced accuracy, specificity decreased by more than 20% compared to the cross-validation, which still indicated a moderate performance. This could be explained by the fact that 31 compounds from the BCRP and 35 compounds from the P-gp test set were out of domain, when local outlier factor algorithm was used for the applicability domain estimation (Table 4) [52]. Using the same approach for OATP1B1 and OATP1B3, indicated a total of 9 outliers and similar decrease in performance. For the remaining test sets no results could have been retrieved due to the low number of data points obtained from ChEMBL28. Nevertheless, a tenfold cross validation was carried out for each transporter dataset indicating performances close to 80% for five out of six transporter datasets, making it a valuable and feasible tool for the prediction of new data related to both ABC and SLC transporters. Additionally, this approach benefits from the model’s ability to be updated and shared in a facile manner using Jupyter Notebook.

**Table 4:** Applicability domain estimation for all six transporter protein test sets

Endpoint	LOF result	
	Compounds In-domain	Compounds Out of domain
Breast cancer resistance protein (BCRP)	156	31
Bile salt export pump (BSEP)	9	1
Organic anion transporting polypeptide 1B1 (OATP1B1)	15	6
Organic anion transporting polypeptide 1B3 (OATP1B3)	14	3
Multidrug resistance associated protein (MRP3)	3	0
P-glycoprotein (Pgp)	201	35

## Conclusion

In this study, we present a JN which enables the user to generate classification models for six transporter proteins (BCRP, BSEP, OATP1B1, OATP1B3, MRP3, P-gp) based on four different classifiers with pre-selected descriptors and without extensive hyperparameter search. In addition, the notebook can further be used to create models for additional transporters as well as retraining of the existing prediction models using pre-defined descriptors as well as hyperparameters with an extended/novel dataset. The JN can be as well used for educational purposes, especially for the ones interested in the creation of predictive ML models for inhibitory activity predictions.

## Data Availability

The Jupyter Notebook as well as relevant files for the model building can be found in our GitHub repository at https://github.com/PharminfoVienna/Retraining_Notebook.

## Abbreviations

BCRP: Breast cancer resistance protein
BSEP: Bile salt export pump
MRP3: Multidrug resistance-associated protein 3
OATP1B1: Organic anion transporting polypeptide 1B1
OATP1B3: Organic anion transporting polypeptide 1B3
P-gp: P-glycoprotein
SMILES: Simplified molecular input line entry system
InChI: International chemical identifier
LOF: Local outlier factor
AUC: Area under the curve
MCC: Matthews correlation coefficient
